# Quantifying Oxygen Levels in 3D Bioprinted Cell-Laden Thick Constructs with Perfusable Microchannel Networks

**DOI:** 10.3390/polym12061260

**Published:** 2020-05-30

**Authors:** Lara Figueiredo, Catherine Le Visage, Pierre Weiss, Jing Yang

**Affiliations:** 1 Regenerative Medicine and Cellular Therapies Group, School of Pharmacy, University of Nottingham, Nottingham NG7 2RD, UK; lara_if@hotmail.com; 2 UMR 1229, RMeS, Regenerative Medicine and Skeleton, Université de Nantes, INSERM, ONIRIS, F-44042 Nantes, France; catherine.levisage@univ-nantes.fr; 3 UFR Odontologie, Université de Nantes, 44042 Nantes, France; 4 Biodiscovery Institute, University of Nottingham, Nottingham NG7 2RD, UK

**Keywords:** 3D bioprinting, microfluidics, microchannels, oxygen, hydrogel, silated-HPMC

## Abstract

The survival and function of thick tissue engineered implanted constructs depends on pre-existing, embedded, functional, vascular-like structures that are able to integrate with the host vasculature. Bioprinting was employed to build perfusable vascular-like networks within thick constructs. However, the improvement of oxygen transportation facilitated by these vascular-like networks was directly quantified. Using an optical fiber oxygen sensor, we measured the oxygen content at different positions within 3D bioprinted constructs with and without perfusable microchannel networks. Perfusion was found to play an essential role in maintaining relatively high oxygen content in cell-laden constructs and, consequently, high cell viability. The concentration of oxygen changes following switching on and off the perfusion. Oxygen concentration depletes quickly after pausing perfusion but recovers rapidly after resuming the perfusion. The quantification of oxygen levels within cell-laden hydrogel constructs could provide insight into channel network design and cellular responses.

## 1. Introduction

Tissue engineering holds promise for the production of replacement tissues and organs to address the current shortage in donated organs [1,2]. However, the fabrication of complex and functional tissues/organs is still very challenging. One of the challenges is to fabricate a functional blood vessel network that can integrate with the host to facilitate nutrient and oxygen transport [3,4]. Diffusion limit of oxygen in vivo is approximately 200 µm, and cells have to reside within this distance from a capillary to survive [5,6]. Furthermore, the interactions between vascular promoting factors, endothelial cells and nerve cells have demonstrated that a microvascularization system helps innervation and tissue formation [7].

Various approaches, including angiogenesis induction by growth factors [8] and engineered vascular-like perfusable networks [9,10,11], have been developed to address the challenge of vascularization. Growth factors, such as vascular endothelial growth factor(VEGF) and basic fibroblast growth factor (bFGF), stimulate the recruitment of endothelial cells [12] and have been shown to improve vascularization after implantation of tissue engineered constructs [8]. However, this strategy requires a relatively long time to establish a fully functional vasculature. During this period, the cells in the constructs rely on the diffusion of oxygen and nutrients from the host, which can comprise cell survival in thick constructs. The mass transportation before the establishment of a vasculature is limited, which suggests the requirement of prevascularization [13]. Other approaches based on oxygen-delivering biomaterials have also been trialed to deliver temporary oxygen bridging before neo-vascularization [14,15]. However, these approaches suffer from drawbacks, such as production of toxic reactive oxygen species and local inflammation. An important advantage of pre-vascularization, over in vivo vascularization induction, is the immediate perfusion of oxygen and nutrients, bypassing the time-lag for vasculature to be formed [16]. Three-dimensional bioprinting has been employed to fabricate pre-vascularized tissue constructs, in which typically a bioink and a sacrificial material are co-printed to form construct. The sacrificial material is removed afterwards to make perfusable channel networks [10,17].

Several elegant methods for 3D printing perfusable constructs with vascular-like microchannels have been reported. Miller et al. have produced a 3D carbohydrate lattice that was dissolved in media after matrix bulk embedding. For hepatocytes seeded constructs, it was shown that the perfused construct with channels had sustained cellular metabolism when compared with gel slabs [9]. Thick constructs (>1 cm), integrating human mesenchymal stem cells (hMSCs), human neonatal dermal fibroblasts (hNDFs), and human umbilical vein endothelial cells (HUVECs) that were perfused for long durations (>6 weeks), have been assembled by coprinting multiple inks at ambient conditions. When actively perfused with osteogenic media, these vascularized cell-laden constructs showed a significantly higher level of calcium phosphate formation deep within the core compared to avascular constructs [18].

The improvement of cell survival and function has been attributed to the increased transport of nutrients, oxygen, and waste products. However, to our knowledge, the oxygen level in bioprinted cell seeded prevascularized constructs is yet to be quantified. Local oxygen quantification will serve as a measurement of the efficacy of the bioprinted channels. In this study, we selected silated-hydroxypropylmethylcellulose (Si-HPMC) hydrogel, a cellulose ether derivative, that has been used in cartilage [19], bone [20] tissue engineering, and was demonstrated to be biocompatible [20,21,22]. Si-HPMC hydrogel crosslinking is promoted by pH neutralization, which makes it a good candidate for bioprinting as it is a cytocompatible process and has a printing time window of about 30 min, during which the gel viscosity increases [23]. In modeling studies, some reports found approximations of the oxygen diffusivity in acellular hydrogels equal to the diffusivity in water. However, previous characterization of the Si-HPMC has shown that these approximations do not apply for all types of hydrogels. Specifically, oxygen diffusivity in a hydrogel with 99% water and 1% Si-HPMC was 12.7% of that in water (2.7 × 10^−9^ m^2^ s^−1^) [24], suggesting the importance of vascularization for constructs of large dimensions.

Herein, a thick structure with perfusable channels and cell-laden Si-HPMC was bioprinted. The oxygen levels within the 3D bioprinted constructs with perfusable channels was quantified, in the presence of cells, using an optical fiber oxygen sensor. This is the first time, to the best of our knowledge, that oxygen concentrations in 3D bioprinted cell seeded constructs with an organized 3D channel architecture were quantified. Our findings show that microchannels alone are not sufficient to maintain sufficient oxygen levels and that perfusion is key for sustaining a high oxygen concentration and cell viability within the constructs.

## 2. Materials and Methods

### 2.1. Solutions Preparation

Hydroxypropylmethylcellulose (HPMC) E4M^®^, obtained from Colorcon (Kent, UK) was silanized in a process previously described to form Si-HPMC [25]. Si-HPMC polymer was dissolved in 0.2 M NaOH aqueous solution and then dialyzed against a 0.09 M NaOH solution with a molecular weight cut off of 6–8 kDa. The viscous solution was autoclaved at 121 °C and kept at room temperature until usage. For the preparation of the hydrogel, one volume of 3 wt % Si-HPMC was mixed with half volume of acidic buffer to achieve a final concentration of 2% Si-HPMC and pH of 7.4. The acidic buffer consisted of a sterile 0.06 M HCl solution with 1.8% NaCl (*w/v*) and 6.2% (*w/v*) HEPES (4-(2-hydroxyethyl) piperazine-1-ethanesulfonic acid). All products were obtained from Sigma (St. Louis, MO, USA).

A 6% (*w*/*v*) gelatin solution was prepared by dissolving gelatin (porcine skin, gel strength ~300 g Bloom (Sigma)) powder in PBS at 60 °C under agitation. After complete dissolution, the solution was autoclaved at 121 °C and stored at 4 °C in aliquots.

### 2.2. Sheep Primary Cells Culture

Sheep primary bone marrow stromal cells (sMSC) were expanded in complete medium which consists of *α*MEM, 10% fetal bovine serum 1% antibiotic/antimycotic, 1% L-glutamine, and 50 µg/mL ascorbic acid. Cells were cultured until 80% confluence and used between passages 2 and 5.

### 2.3. 3D Bioprinting of Constructs

Si-HPMC was mixed with the acidic buffer, as described in Section 2.1, using two syringes connected by a Luer Lock. sMSC were trypsinized, centrifuged and the resulting pellet was resuspended in 100 µL of complete medium. Twenty-five minutes after Si-HPMC neutralization, 24 million sMSC were added to 3 mL of hydrogel with a micropipette, without creating air bubbles. We selected this cell density (8 million/mL) as our previous study has shown that this cell density caused significant cell death in bulk Si-HPMC hydrogels without microchannels [24]. Cells were homogenized in the hydrogel through mixing in two syringes connected by a Luer Lock. The bioink (sMSC/Si-HPMC) was then transferred to a syringe barrel and printed with a 27-gauge needle at room temperature. For the bioprinting of constructs with microchannels, gelatin 6% was co-printed through a 30-gauge needle at 10 °C, using a cooling system installed on the 3D printer (3DDiscovery, RegenHU, Villaz-Saint-Pierre, Switzerland). The two materials were printed in a glass round coverslip using the same extrusion 3D printer. The BioCAD software on the printer was used to design the 3D constructs with and without microchannels. For the bioprinting of the construct without microchannels, only Si-HPMC was bioprinted in a total of 10 layers. For the bioprinting of the construct with microchannels, the two bottom layers and the two top layers were composed of Si-HPMC only. Six intermediary layers combined Si-HPMC and gelatin materials. Si-HPMC strands were 0.6 mm wide, and gelatin strands were 0.3 mm wide. For each intermediary layer, one layer of Si-HPMC and two superimposing layers of gelatin were printed with a total height of 0.4 mm per layer. Total dimensions of the constructs were 9 × 9 × 4 mm^3^ (length × width × height).

Constructs on top of coverslips were transferred to a Petri dish enclosed in a second adapted Petri dish that allowed the attachment of the adaptors of the perfusion tubbing into the channels when necessary. 

### 2.4. Observation of Printed Micro-Channels

Constructs with microchannels and without cells were embedded in OCT compound immediately after printing and frozen at 80 °C overnight. The frozen samples were cryosectioned at a thickness of 200 µm using a cryo-microtome (Leica CM1900, Nussloch, Germany). Cryosections were incubated at 37 °C in PBS for total removal of gelatin. Trypan blue was added to provide contrast, samples were observed with a Leica DM IRB microscope, and photographs were taken with a QImaging QICAM camera.

### 2.5. Oxygen Concentration Measurements

Oxygen partial pressure in the 3D printed constructs was monitored at 24 h, at different depths using a micromanipulator (Eppendorf TransferMan NK2, Hamburg, Germany), with a needle type oxygen microsensor (PreSens, Regensburg, Germany). The microsensor, a retractable, 230 µm diameter optic fiber, allows real-time oxygen measurements, without oxygen consumption, through dynamic fluorescence quenching, with data reported to an Oxy-4 transmitter. The sensitivity of the oxygen sensor is 0.03%. This sensor was validated in our previous work by measuring oxygen diffusivity in water [24]. Since oxygen diffusion is a temperature dependent phenomenon, all measurements were performed at 37 °C. 

### 2.6. Cell Viability

Cell viability measurements were performed at day 0, 1, 7, and 14 using a Live/Dead assay kit (Invitrogen, Carlsbad, CA, USA). Briefly, hydrogels were washed twice with *α*MEM to remove esterase activity of the serum-supplemented growth medium and then incubated with 2µM calcein-AM solution and 4 µM Ethidium homodimer-1 in αMEM for 30 min at room temperature. Samples 500 µm × 500 µm × 200 µm were then collected from the center of the constructs and imaged at 3 different locations with a fluorescence microscope (Leica, GmbH, Wetzlar, Germany). Images were analyzed with ImageJ to count green and red cells. Results are presented as a percentage of live cells (mean value ± SEM, *n* = 3 independent experiments).

### 2.7. Perfusion of the Printed Constructs

Gelatin was first removed by incubating the constructs at 37 °C for one hour in complete *α*MEM. Silicone tubing was connected through a 22-gauge needle to the inlet of the channel network guided through an in-house printed PCL adaptor. Constructs were immersed in culture medium that was pumped into the constructs using a peristaltic pump at a 500 µL/min rate (Watson-Marlow, Marlow, UK). For the perfusion cycles, the pump was stopped, and the tubbing was kept attached, while performing oxygen continuous measurements, until oxygen levels reached stable readouts. The pump was reinitiated until initial oxygen levels were recovered. 

### 2.8. Statistical Methods

Statistical significance was determined by one-way ANOVA with a post hoc Tukey test using GraphPad 5 (GraphPad Software Inc., La Jolla, CA, USA) statistical analysis.

## 3. Results

### 3.1. 3D Printing and Characterization of the Perfusable Constructs

Previous results showed that Si-HPMC hydrogel starts crosslinking after pH neutralization, gelation point occurs at 30 min [23], and crosslinking continues at room temperature over several hours. In this study, cells were mixed with the polymer solution 5 min before the gel point to minimize mechanical shear stress. Three-dimensional printing was carried out using the bioink (sMSC/Si-HPMC) after the gelling point to form a stable construct with designed dimensions of 9 × 9 × 4 mm^3^ (length × width × height).

Better printability was achieved by autoclaving gelatin at 120 °C to reduce its molecular weight, turning it less brittle, and thinner strands of gelatin could be printed at 10 °C. The thermally reversible gelation of gelatin allowed for the removal of this material at 37 °C, which is convenient when working with cell seeded constructs [26].

**Figure 1:**
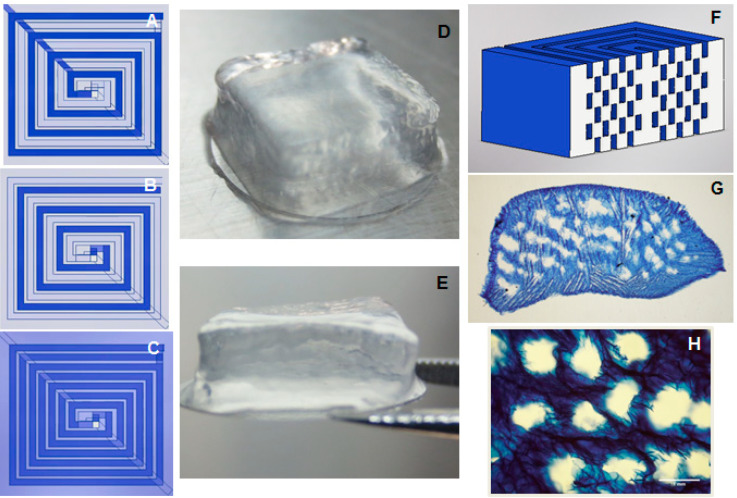
Three-dimensional printed Si-HPMC constructs with microchannels. (**A**) Design of the first of the odd layers of the construct, where darker line represents gelatin printed strands, and lighter lines represent Si-HPMC strands. (**B**) Design of even layers of the construct. (**C**) Representation of the top view of the construct morphology of the 3D bioprinted construct of cell-laden Si-HPMC. Printed constructs with microchannels (**D**) before and (**E**) after gelatin removal (9 × 9 × 4 mm). (**F**) Representation of a cross section of the channeled construct (without bottom and top Si-HPMC-only layers). (**G**) Optical image of the transversal slice of the stained construct exhibiting microchannels after gelatin removal. (**H**) A zoomed-in part of the transversal slice. Si-HPMC: silanized hydroxypropylmethylcellulose.

The schematic representation in Figure 1A–C shows the design of microchannels embedded in cell-laden hydrogels. The construct was bioprinted layer-by-layer, with gelatin strands in subsequent layers being printed on top of the bioink strands, in order to avoid superimposing and conserve the integrity of the thick construct. Gelatin strands superimposed at a single point at the center of the constructs to allow the connection of channels in all the layers.

After 3D printing, constructs were immersed in PBS for one hour at 37 °C to remove the gelatin. The structure of the construct was not affected by the removal of gelatin nor by the large proportion of microchannels, as shown in Figure 1D–E (before and after gelatin removal, respectively). A construct cross section shows the distribution of microchannels within the 10-layer construct with six layers of microchannels (Figure 1G–H). It appears that some microchannels may have fused together (Figure 1G,H). However, this could be due to deformation caused by the cryo-sectioning process and the thinness of the sectioned hydrogel slice.

In order to show that microchannels at all layers were interconnected, the construct was perfused with a dye solution. It was observed that the dye solution occupied all the volume of the hollow microchannels (MOVIE 1 in Supplementary Materials), suggesting that there was no obstruction of microchannels and that all channels were in fact interconnected.

### 3.2. Oxygen Diffusion in the Different Constructs

**Figure 2:**
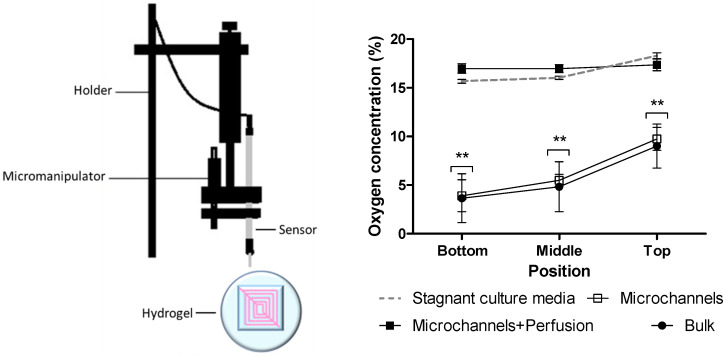
**(Left**) Schematic of the apparatus for measuring oxygen concentrations. (**Right**) Oxygen levels at different heights of the constructs with and without perfusion, 24 h after bioprinting of the constructs. “Microchannels” refers to the constructs with microchannels and without perfusion; “Microchannels+Perfusion” refers to the constructs with microchannels and perfusion; “Bulk” refers to the constructions without channels. All constructs were seeded with a cell density of 8 million cells/mL (*n* = 3, data represents mean ± SEM). ** *p* < 0.01 when compared to culture media.

According to previous results, oxygen concentration at the center of similar constructs without microchannels was shown to be reduced after 6 h of incubation [24], and, at 24 h, differences were expected for the different types of constructs. After one day of incubation, oxygen concentration was measured at the top, middle, and bottom levels of three types of constructs: bulk constructs without microchannels, as well as channeled constructs with and without perfusion. Due to the loss in mass after gelatin microchannels evacuation, different constructs did not conserve the same measurements after incubation. In order to establish reference points for the comparison between constructs, top, middle, and bottom points were defined. Top was defined at the height where the oxygen sensor first touched the construct with the guidance of a micromanipulator. Bottom was defined as the point where the oxygen sensor reached the surface where the construct was placed. The difference between top and bottom height was established as height of the construct. The middle was then calculated for each construct as the half the height. Replicated measurements were done in three central and independent points and always restarted from top to bottom, in order to exclude interference by oxygen consumption by the cells while measurements took place. All constructs were seeded at a cell density of 8 million cells/mL and all constructs were completely immersed in culture media except for the bottom side that was not directly exposed to the media. With a calculated 20% density of microchannels, it is not possible to distinguish between readings done with the oxygen sensor at the interior of the microchannels or at the interstitial space between the microchannels. In the absence of perfusion, oxygen concentration decreases from the top to the bottom of the constructs, independently of the presence of microchannels (Figure 2). Without perfusion, bulk constructs showed similar oxygen concentrations at the three different positions compared to constructs with microchannels. With perfusion, the three positions at different heights showed similar high oxygen concentrations, suggesting the importance of perfusion for effective mass transport. Oxygen concentrations in constructs with microchannels and perfusion were similar to that in stagnant pure media (18% ± 0.35).

**Figure 3:**
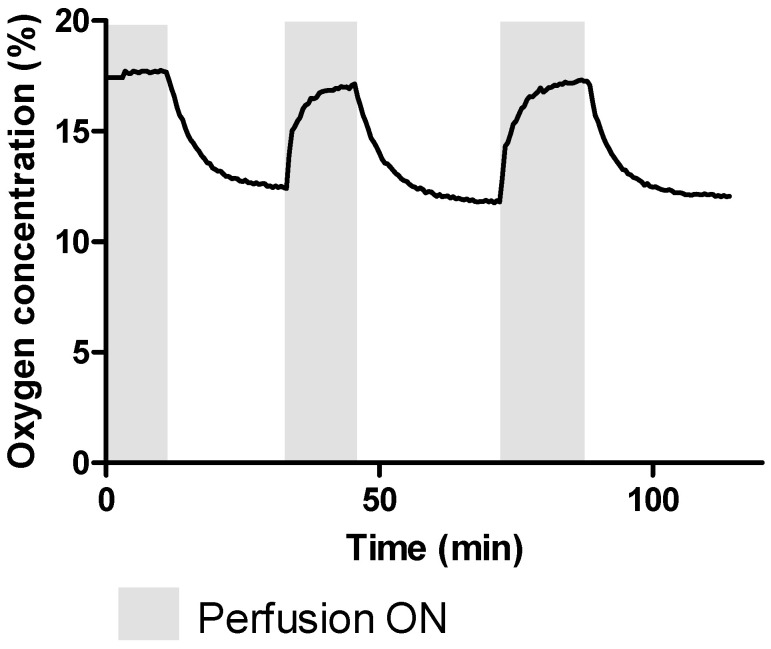
Representative oxygen concentration in the center of a Si-HPMC 2% construct with microchannels seeded with a cell density of 8 million cells/mL and subject to perfusion cycles until recovery of oxygen concentration.

When perfusion was turned off, an exponential drop in oxygen concentration took place. The oxygen concentration in the middle of the construct dropped approximately 5%, from 17% to 12%, in less than 20 min (Figure 3). After perfusion resumed, the oxygen concentration recovered to 17% in 13 min on the first recovery and 16 min on the second recovery. The initial recovery was rapidly followed by a more graduate increase, as can be seen in the last part of the curve of the grey areas in Figure 3.

### 3.3. Cell Viability

**Figure 4:**
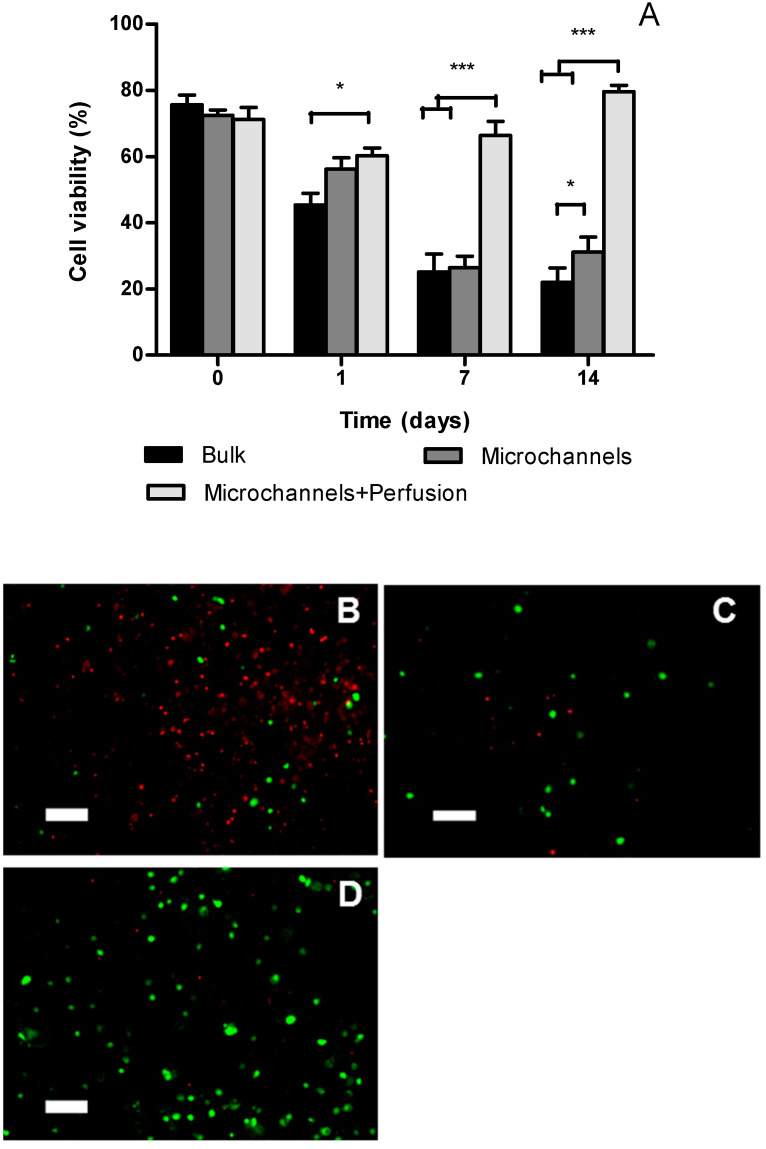
Cell viability at the geometric center of Si-HPMC 2% constructs 9 × 9 × 4 mm^3^ (length × width × height) seeded with 8 million cells/mL was assessed with Live/Dead assay using confocal microscopy. (**A**) Cell viability at 0, 1, 7, and 14 days after bioprinting. Values are mean ± SEM (*n* = 3). (**B**–**D**): Confocal microscopy images of Live (green) and Dead (red) cells in the center of the constructs 14 days after bioprinting (**B**: Bulk construct; **C**: with microchannels; **D**: with microchannels and perfusion). scale bar = 100 µm. * *p* < 0.05; *** *p* < 0.001.

Cell viability was assessed at four different time points in samples recovered from the geometric center of each cellular construct 9 × 9 × 4 mm^3^ (length × width × height). Figure 4A shows that cell viability was between 76% and 71% immediately after bioprinting for the bulk constructs and constructs with microchannels, respectively. At day 1, cell viability at the center of the bulk constructs was significantly lower than those in the construct with micro-channels and perfusion. However, there was no statistical difference between channeled constructs with and without perfusion. At day 7 and 14, the viabilities in constructs without perfusion dropped significantly compared to day 1. In contrast, the viability in perfused constructs steadily increased from day 1 to day 14 and was significantly higher than non-perfused constructs at day 7 and day 14.

## 4. Discussion

The incorporation of vascular-like channels is expected to facilitate the transport of oxygen and nutrients in thick constructs. Although various approaches have been developed to prevascularize thick cell-laden constructs, as far as the authors know, the oxygen levels with these constructs have not been quantified before. One of the hydrogels used in our bioprinted perfusable constructs was Si-HPMC, which is cyto- and biocompatible, as shown in previous works [20,22]. Being a self-setting hydrogel, Si-HPMC does not require UV curing, eliminating the low cell viability after repeated UV exposure required for 3D printing large-size constructs [27]. Two percent Si-HPMC shows a gelling point 30 min after pH neutralization at room temperature, which allows a convenient time window for cell encapsulation under cytocompatible conditions before the gel becomes too viscosity for homogenous cell mixing. Previous work [24] has established that, with a cell density 8 M cells/mL in 2% Si-HPMC constructs, cell viability is reduced to less than 1% at 72 h after seeding concomitantly with a complete depletion of oxygen in the center of the constructs (10 mm high). Therefore, we selected this cell density in this study for comparison. The perfusion of micro-channels in the bioprinted thick constructs supported the survival of cells within the central region with an approximate cell viability of 80% after 14 days of culture, which demonstrated the importance of perfusion for maintaining cell viability in thick constructs. The difference in oxygen concentration was approximately 12% between the central region of the bulk hydrogel and that of the perfused counterpart. This difference has caused a significantly lower cell viability in the bulk hydrogel.

The quality of the sectioned slices was compromised by the sample preparation process of cryosectioning. Consequently, the shape and distribution of the channels may have been distorted. However, we have demonstrated the connectivity of these channels by perfusing a dye through the printed channels. There are other non-destructive methods, such as perfusion of a dye that can be visualized in microCT, that can be utilized to assess the channels. However, we believe that extra characterization does not affect the main finding of this work, which is the importance of perfusion and local measurement to ensure appropriate oxygen concentrations.

The flow rate of 0.5 mL/min was selected as it allowed quick perfusion of the 3D printed constructs. The value of this flow rate is smaller than reported blood flow (1.2–4.8 mL/min in veins) [28]. A higher flow rate may shorten the oxygen concentration recovery time which is 14.2 min using the flow rate of 0.5 mL/min. However, it will not increase the maximum oxygen concentration as the perfused constructs showed similar oxygen level compared to medium. The recovery of oxygen concentration was not linear with an initial fast increase followed by a slow increase before reaching a steady state. The time to reach 90% of the maximum oxygen concentration was 2.3 min, which was only a small fraction of the total recovery time.

We demonstrated the kinetics of the recovery of oxygen concentration after perfusion. The local oxygen concentrations in human body are intricately controlled. Different organs or even different locations of an organ can experience different oxygen tensions [29]. Oxygen delivery depends on the metabolic demand and functional status of each organ. One interesting future work would be to utilize this kind of kinetic data to deduce the metabolic status of the cells. This data could then be compared to physiological data to infer the difference between in vitro and in vivo conditions, which may assist in developing better in vitro models that recapitulate tissues and organs.

We used an invasive method to measure oxygen concentration. The advantage of this method is that it is relatively easy to read the oxygen levels at different positions. With the assistance of a micromanipulator, the position from which oxygen was measured was precisely controlled. Others have used fluorescence-based methods to probe oxygen concentration [30]. The advantage of this method is that it is less invasive though micro-probes need to be embedded in the hydrogels. The disadvantage of this method is that it is a light-based technique meaning the transparency, and thickness of the hydrogel can limit its application.

The addition of layers of hydrogel and gelatin with no gaps to subsequent layers provides a plane surface for the next layer, granting stability to the construct, and no deformation was observed as in previous cases [31]. The high density of channels allows cells to be in close contact with perfused oxygen, not further than 300 µm.

Bulk constructs and constructs with microchannels without perfusion showed similar levels of oxygen. This shows that perfusion is necessary for the maintenance of oxygen levels in the cell-laden constructs. Simple diffusion of oxygen into thick constructs is not sufficient to compensate for the oxygen consumed by the cells at high density. That the oxygen levels were not influenced by the presence of micro-channels in the absence of perfusion suggests the importance of connecting bioprinted perfusable constructs with the host’s vasculature when grafting in vivo.

Our work has important implications for 3D bioprinting of functional vascularized organs. The body of work on 3D printing of vascularized tissues and organs is rising rapidly. Much effort has been focused on creating a perusable channel network within thick cellular hydrogels. Our work here has demonstrated the importance of perfusion in maintaining high oxygen levels in thick cellular hydrogels, which means that the ability of a built-in vasculature in engineered organs anastomosing to host vasculature is critical. We believe this will be the next challenge in vascularized 3D bioprinted organs which allow the engineered vasculature to be connected to host vasculature in surgeries facilitating immediate mass transportation without causing significant cell death.

## 5. Conclusions

Cellular constructs with embedded 3D interconnected micro-channel networks were successfully bioprinted using bone marrow stromal cell-laden Si-HPMC and sacrificial gelatin that can easily be removed at 37 °C. Oxygen concentrations at different positions were measured in the cell-laden constructs with and without perfusable channels. We have shown that perfusion is key for maintaining a high oxygen concentration within the constructs. Without perfusion the oxygen concentration within channeled constructs was similar to that in solid constructs. When perfusion was turned off, the oxygen dropped 6% in less than 20 min and recovered 17% in 14 min after perfusion was turned on. The quantification of oxygen content in bioprinted perfusable constructs can help offer insight into the channel design and explain cellular responses.

## Supplementary Materials

The following are available online at https://www.mdpi.com/2073-4360/12/6/1260/s1, Video S1: Perfusion of the 3D printed construct after removal of the sacrificial ink (8 × 8 × 6 mm).
